# 联合社会人口学因素及临床因素预测初发慢性髓性白血病慢性期患者治疗反应及结局

**DOI:** 10.3760/cma.j.issn.0253-2727.2022.01.011

**Published:** 2022-01

**Authors:** 小帅 张, 亚溱 秦, 悦云 赖, 红霞 石, 晓军 黄, 倩 江

**Affiliations:** 北京大学人民医院、北京大学血液病研究所、国家血液系统疾病临床医学研究中心 Peking University People's Hospital, Peking University Institute of Hematology, National Clinical Research Center for Hematologic Disease, Beijing 100044, China

**Keywords:** 白血病，髓系，慢性, 治疗结果, 社会学因素, 临床因素, Leukemia, myeloid, chronic, Treatment outcome, Predictive models, Clinical co-variates

## Abstract

**目的:**

探讨影响初发慢性髓性白血病慢性期（CML-CP）治疗反应及结局的社会人口学因素及临床因素，并联合社会人口学因素及临床因素分别建立针对治疗反应及结局新的预测模型。

**方法:**

回顾性分析2006年1月至2020年12月在北京大学人民医院确诊的≥18岁初诊接受酪氨酸激酶抑制剂（TKI）一线治疗、具有完整社会人口学及临床资料的CML-CP连续病例。应用Cox回归模型探索治疗反应和结局的独立影响因素，治疗反应包括完全细胞遗传学反应（CCyR）、主要分子学反应（MMR）、分子学反应4（MR^4^）、分子学反应4.5（MR^4.5^），结局包括无失败生存（FFS）、无进展生存（PFS）、总生存（OS）、CML相关生存（CML-OS）。

**结果:**

研究共纳入1414例以伊马替尼（1176例）、尼洛替尼（170例）或达沙替尼（68例）为一线治疗的成人CML-CP患者，中位年龄40（18～83）岁，男性873例（61.7％）。多因素分析显示，受教育水平低（*P*值<0.001～0.070）和ELTS评分中/高危（*P*值<0.001～0.009）是较低的细胞遗传学、分子学反应获得率以及各种不良治疗结局的共同影响因素。此外，男性、农村户籍以及初诊时WBC≥120×10^9^/L、HGB<115 g/L和一线伊马替尼治疗与较低的细胞遗传学反应和（或）分子学反应获得率均显著相关；单身、离异或丧偶、农村户籍以及初诊时WBC≥120×10^9^/L、HGB<115 g/L和伴有合并症与较低的FFS、PFS、OS和（或）CML-OS率显著相关。联合社会人口学因素及临床因素分别建立针对治疗反应、治疗失败及疾病进展以及生存的预测模型，根据上述预测模型将整体患者分为各风险亚组，亚组间治疗反应及结局差异均具有统计学意义（*P*值均<0.001）。

**结论:**

除临床因素外，社会人口学因素与CML-CP患者治疗反应及结局密切相关。联合社会人口学因素与临床特征可较好预测患者的治疗反应及结局。

## Abstract (en)

**Objective:**

To explore the impacts of socio-demographic and clinical co-variates on treatment responses and outcomes in patients with chronic myeloid leukemia in the chronic phase (CML-CP) receiving tyrosine kinase inhibitor (TKI) and identified the predictive models for them.

**Methods:**

Data of newly diagnosed adult patients with CML-CP receiving first-line TKI and having complete socio-demographic data and clinical information were reviewed. Cox model was used to identify the independent variables associated with complete cytogenetic response (CCyR), major molecular response (MMR), molecular response 4 (MR^4^) and molecular response 4.5 (MR^4.5^), as well as failure-free survival (FFS), progression-free survival (PFS), overall survival (OS) and CML-related OS.

**Results:**

A total of 1414 CML-CP patients treated with first-line imatinib (*n*＝1176), nilotinib (*n*＝170) or dasatinib (*n*＝68) were reviewed. Median age was 40 (18–83) years and 873 patients (61.7％) were males. Result of the multivariate analysis showed that lower educational level (*P*<0.001−0.070) and EUTOS long-term survival intermediate or high-risk (*P*<0.001−0.009) were significantly associated with lower cumulative incidences of CCyR, MMR, MR^4^ and MR^4.5^, as well as the inferior FFS, PFS, OS and CML-related OS. In addition, those who were males, from rural households, had white blood cells (WBC) ≥120×10^9^/L, hemoglobin (HGB) <115 g/L and treated with first-line imatinib had significantly lower cumulative incidences of cytogenetic and/or molecular responses. Being single, divorced or widowed, having, rural household registration, WBC≥120×10^9^/L, HGB<15 g/L, and comorbidity (ies) was significantly associated with inferior FFS, PFS, OS, and/or CML-related OS. Thereafter, the patients were classified into several subgroups using the socio-demographic characteristics and clinical variables by cytogenetic and molecular responses, treatment failure and disease progression, as well as overall survival and CML-related OS, respectively. There were significant differences in treatment responses and outcomes among the subgroups (*P*<0.001).

**Conclusion:**

Except for clinical co-variates, socio-demographic co-variates significantly correlated with TKI treatment responses and outcomes in CML-CP patients. Models established by the combination of independent socio-demographic and clinical co-variates could effectively predict the responses and outcome.

大部分慢性髓性白血病慢性期（CML-CP）患者经酪氨酸激酶抑制剂（TKI）治疗可获得良好的细胞遗传学、分子学反应以及生存结局，预期寿命近乎常人，但仍有约20％的患者在TKI治疗中出现治疗失败、疾病进展甚至死亡[1]–[4]。为了更好地预测TKI治疗反应和结局，数种预后积分系统相继被提出，如Sokal、Hasford、EUTOS和ELTS评分等[5]–[8]。近年来，研究显示ELTS评分在预测患者CML相关死亡和总生存（OS）期上优于其他预后积分系统，在预测治疗反应方面也被证实具有优势[9]–[14]。不少研究报道，除了ELTS评分，患者社会人口学特征包括性别、户籍、受教育水平和婚姻状态等也显著影响CML患者的治疗反应和结局[15]–[22]。目前，国内外尚无联合ELTS评分和社会人口学特征综合预测患者治疗反应及结局的研究。为此，我们回顾性分析本中心病例资料，联合ELTS评分与社会人口学因素，探索新的初诊CML-CP患者治疗反应及结局预测模型。

## 病例与方法

一、病例

回顾性分析2006年1月至2020年12月在北京大学人民医院确诊、年龄≥18岁、确诊CML后6个月内接受TKI作为一线治疗的CML-CP连续病例。收集初诊患者社会人口学资料（包括年龄、性别、受教育水平、户籍和婚姻状态）、全血细胞计数、外周血原始细胞及嗜碱性粒细胞比例、初诊染色体核型、合并症、ELTS评分、TKI种类和剂量、治疗反应和结局。

二、诊断分期

参照MD Anderson癌症中心CML诊断和分期标准[23]。

三、治疗

根据患者的年龄、疾病危险度、合并症、合并用药以及经济情况等因素，由患者和医师共同决定一线TKI用药选择，初始采用标准剂量（伊马替尼400 mg每日1次、尼洛替尼300 mg每日2次或达沙替尼100 mg每日1次），治疗中根据治疗反应和（或）不良反应等调整TKI剂量或类型。

四、监测

参照2006、2009、2013及2020年欧洲白血病网（ELN）指南[24]–[27]进行治疗反应监测。

1. 血液学：每1～2周进行血细胞计数和分类，直至达到完全血液学反应（CHR）后，每3个月进行1次。

2. 细胞遗传学：每3个月采用染色体G显带法进行骨髓细胞遗传学分析，直至达到完全细胞遗传学反应（CCyR）后，每12～18个月进行1次。

3. 分子学：每3个月通过实时定量聚合酶链反应（RQ-PCR）检测外周血BCR-ABL转录本水平，以ABL为内参基因，ABL拷贝数>32 000。直至获得主要分子学反应（MMR）后，每3～6个月进行1次。

五、评估指标

治疗反应和结局分析均采用意向性治疗分析法（ITT）。

1. 治疗反应：根据2020年ELN指南[27]，定义CHR、CCyR、MMR、分子学反应4（MR^4^）和分子学反应4.5（MR^4.5^）。

2. 治疗结局：①无治疗失败生存（FFS）期：从开始TKI治疗至治疗失败或末次随访的时间；②无疾病进展生存（PFS）期：从开始TKI治疗至进展到加速期/急变期、死亡或末次随访的时间；③总生存（OS）期：从开始TKI治疗至死亡或末次随访的时间；④CML相关生存（CML-OS）期：从开始TKI治疗至CML相关死亡（CML进展为加速期/急变期后死亡）或末次随访的时间[7]。所有结局观察均删失至造血干细胞移植。

六、随访

采用门诊或电话联系的方式进行随访，随访截止时间为2021年5月。

七、统计学处理

患者基线信息包括社会人口学资料及临床特征采用描述性统计学分析。组间比较时，连续变量进行Kruskal-Wallis非参数检验，分类变量进行卡方检验。针对连续变量包括WBC、HGB及外周血嗜碱性粒细胞比例，采用ROC曲线确定其预测不良结局的最佳截断值。对于累积治疗反应获得率采用竞争风险模型分析，其竞争风险为移植或死亡，并应用Fine-Grey检验进行组间比较；对于结局采用Kaplan-Meier生存曲线分析，并应用Log-rank检验进行组间比较；将患者社会人口学因素和初诊时临床参数进行单因素分析时，*P*<0.2的变量纳入Cox回归模型进行多因素分析。根据多因素分析结果，将影响所有治疗反应及结局的共同因素作为分组变量，对所有患者重新进行危险度评分。*P*<0.05为差异有统计学意义。分别采用SPSS 22.0、R 4.0.2及GraphPad Prism 8软件进行统计分析、绘图。

## 结果

1. 患者特征：2006年1月至2020年12月，共收集到1840例≥18岁CML-CP连续性病例资料，排除社会人口学信息不全216例、关键临床信息缺失（包括初诊时全血细胞计数及分类、初诊染色体核型等）84例、不规律随访或失访126例，最终本研究纳入具有完整社会人口学信息及临床特征资料的1414例接受伊马替尼（1176例）或二代TKI（238例，包括尼洛替尼170例和达沙替尼68例）作为一线治疗的成人CML-CP患者。中位年龄40（18～83）岁，男性873例（61.7％）。ELTS评分低危942例（66.6％）、中危352例（24.9％）、高危120例（8.5％）。受教育水平≤初中355例（25.1％）、高中312例（22.1％）、≥大学747例（52.8％）。城镇户籍993例（70.2％），农村户籍421例（29.8％）。未婚253例（17.9％），已婚1107例（78.3％），离异或丧偶54例（3.8％）。

与一线伊马替尼治疗人群相比，一线二代TKI治疗人群更年轻（*P*<0.001），初诊时脾大（*P*<0.001）和ELTS中、高危患者比例更高（*P*<0.001），WBC（*P*＝0.002）和PLT（*P*＝0.048）水平更高，HGB水平更低（*P*<0.001），外周血嗜碱性粒细胞比例更高（*P*＝0.001），外周血和骨髓原始细胞比例（*P*<0.001）、高危附加染色体异常（*P*<0.001）和服用原研药（*P*<0.001）患者比例更高，两组间性别、受教育水平、婚姻状态、户籍以及诊断到开始TKI治疗时间的差异均无统计学意义（表1）。

**表1:** 1414例初发慢性髓性白血病慢性期患者基线特征

变量	总体人群（1414例）	一线伊马替尼（1176例）	一线二代TKI（238例）	统计量（*χ*^2^/*t*）	*P*值
年龄［岁，*M*（范围）］	40（18～83）	41（18～83）	35（18～73）	4.730	< 0.001
男性［例（％）］	873（61.7）	729（62.0）	144（60.5）	0.185	0.667
受教育水平［例（％）］				1.223	0.542
初中及以下	355（25.1）	301（25.6）	54（22.7）		
高中	312（22.1）	261（22.2）	51（21.4）		
大学及以上	747（52.8）	614（52.2）	133（55.9）		
婚姻状态［例（％）］				4.605	0.100
未婚	253（17.9）	201（17.1）	52（21.8）		
已婚	1107（78.3）	933（79.3）	174（73.1）		
离异/丧偶	54（3.8）	42（3.6）	12（5.0）		
户籍［例（％）］				0.238	0.626
城镇	993（70.2）	829（70.5）	164（68.9）		
农村	421（29.8）	347（29.5）	74（31.1）		
ELTS评分［例（％）］				19.648	< 0.001
低危	942（66.6）	806（68.5）	136（57.1）		
中危	352（24.9）	286（24.3）	66（27.8）		
高危	120（8.5）	84（7.1）	36（15.1）		
诊断到开始TKI治疗时间［月，*M*（范围）］	0.5（0～6.0）	0.5（0～6.0）	0.4（0～6.0）	0.124	0.682
脾脏可触及肿大［例（％）］	724（51.2）	568（48.3）	156（65.5）	20.235	< 0.001
WBC［×10^9^/L，*M*（范围）］	116.7（2.4～754.7）	109.8（3.2～723.7）	152.7（2.4～754.7）	−3.156	0.002
<120×10^9^/L［例（％）］	723（51.1）	624（53.1）	99（41.6）		
≥120×10^9^/L［例（％）］	691（48.9）	552（46.9）	139（58.4）		
HGB［g/L，*M*(范围)］	116.0（28.0～180.0）	117.0（28.0～180.0）	111.0（57.0～167.0）	4.155	< 0.001
<115 g/L［例（％）］	680（48.1）	538（45.7）	142（59.7）		
≥115 g/L［例（％）］	734（51.9）	638（54.3）	96（40.3）		
PLT［×10^9^/L，*M*（范围）］	410.5（36.0～3707.0）	405.0（36.0～3707.0）	443.0（69.2～2887.0）	−1.981	0.048
外周血嗜碱性粒细胞比例［％，*M*（范围）］	5.0（0～19.0）	4.0（0～19.0）	5.0（0～19.0）	−3.185	0.001
<10％［例（％）］	1251（88.5）	1050（89.3）	202（84.5）		
≥10％［例（％）］	163（11.5）	126（10.7）	37（15.5）		
外周血原始细胞比例［％，*M*（范围）］	1（0～14）	1（0～14）	1（0～14）	−3.677	< 0.001
骨髓原始细胞比例［％，*M*（范围）］	2.0（0～13.0）	2.0（0～13.0）	2.0（0～13.0）	−3.162	< 0.001
有合并症［例（％）］	529（37.4）	447（38.0）	82（34.5）	1.609	0.301
高危附加染色体异常［例（％）］	39（2.8）	24（2.0）	15（6.3）	1263.840	< 0.001
初始TKI类型［例（％）］				93.369	< 0.001
原研药	905（64.0）	689（58.6）	181（76.1）		
仿制药	509（36.0）	487（41.4）	57（23.9）		

注：TKI：酪氨酸激酶抑制剂

截至末次随访日期，一线伊马替尼治疗患者中，229例（19.5％）因治疗失败、31例（2.6％）因不耐受、12例（1.0％）因追求更佳疗效而换为二代TKI，6例（0.5％）因疾病进展接受移植，14例（1.2％）因TKI耐药进入临床试验，9例（0.8％）追求无治疗缓解（TFR）而停药，其余875例（74.4％）仍服用伊马替尼。一线二代TKI治疗患者中，6例（2.5％）因TKI耐药进入临床试验，8例（3.4％）因经济原因、10例（4.2％）因不耐受换为伊马替尼，4例（1.7％）因疾病进展接受移植，4例（1.7％）为追求TFR而停药，其余206例（86.6％）仍服用尼洛替尼（151例，63.4％）或达沙替尼（55例，23.2％）。

2. TKI治疗反应及其影响因素分析：全部1414例患者TKI治疗中位时间50（3～193）个月，累计获得CHR 1363例，CCyR 1273例，可评估获得CCyR时间者1227例。在转录本类型b2a2和（或）b3a2的患者中，累计获得MMR 1043例、MR^4^ 554例、MR^4.5^ 501例，可评估获得时间者分别为1023例、531例和485例。总体人群7年累积CCyR、MMR、MR^4^以及MR^4.5^获得率分别为96.8％、85.4％、62.7％、49.5％。

为预测患者TKI治疗反应，运用ROC法对连续变量包括初诊时WBC、HGB、外周血嗜碱性粒细胞比例选取最大约登指数所对应值为截断值，取整数后即为WBC 120.0×10^9^/L，HGB 115 g/L，外周血嗜碱性粒细胞比例10％。

为识别与治疗反应相关的影响因素，对性别、ELTS评分、婚姻状态、户籍、受教育水平、初诊时WBC、HGB、外周血嗜碱性粒细胞比例、有无根据ELN2020指南定义的高危附加染色体异常[27]、有无合并症、一线TKI种类、原研或仿制药进行单因素分析。多因素分析结果显示，男性、受教育水平低、初诊时WBC≥120×10^9^/L、HGB<115 g/L、ELTS评分中/高危和一线伊马替尼治疗与较低的细胞遗传学反应和分子学反应获得率均显著相关，农村户籍还与较低的CCyR获得率显著相关（表2）。

**表2:** 慢性髓性白血病慢性期患者治疗反应的多因素分析结果

变量	CCyR		MMR		MR^4^		MR^4.5^	
	*HR*（95％*CI*）	*P*值	*HR*（95％*CI*）	*P*值	*HR*（95％*CI*）	*P*值	*HR*（95％*CI*）	*P*值
女性	1.1（1.0～1.3）	0.076	1.3（1.1～1.5）	<0.001	1.2（1.0～1.5）	0.032	1.3（1.1～1.6）	0.002
婚姻状态								
未婚（参考组）								
已婚								
离异/丧偶								
农村户籍	0.9（0.7～1.0）	0.034						
受教育水平		0.011		<0.001		0.017		0.070
大学及以上（参考组）								
高中	0.8（0.7～0.9）	0.003	0.7（0.6～0.9）	<0.001	0.8（0.6～1.0）	0.027	0.8（0.6～1.0）	0.036
初中及以下	0.9（0.8～1.1）	0.520	0.7（0.6～0.8）	<0.001	0.8（0.6～1.0）	0.019	0.8（0.7～1.0）	0.132
ELTS评分		<0.001		<0.001		0.003		0.009
低危（参考组）								
中危	0.8（0.7～0.9）	0.003	0.7（0.6～0.8）	<0.001	0.7（0.5～0.9）	0.002	0.7（0.5～0.9）	0.002
高危	0.6（0.4～0.7）	<0.001	0.5（0.4～0.7）	<0.001	0.6（0.4～1.0）	0.048	0.7（0.5～1.1）	0.161
WBC≥ 120×10^9^/L	0.8（0.7～0.9）	0.008	0.7（0.6～0.8）	<0.001	0.5（0.4～0.7）	<0.001	0.4（0.3～0.5）	<0.001
HGB≥ 115 g/L	1.3（1.1～1.6）	<0.001	1.3（1.1～1.5）	0.001	1.3（1.0～1.6）	0.025	1.1（1.0～1.3）	0.018
Ba≥ 10％								
有合并症								
高危附加染色体异常								
服用仿制药								
一线二代TKI治疗	1.5（1.3～1.8）	<0.001	1.4（1.1～1.6）	0.001	1.3（1.1～1.6）	0.004	1.3（1.0～1.7）	0.032

注：CCyR：完全细胞遗传学反应；MMR：主要分子学反应；MR^4^：分子学反应4；MR^4.5^：分子学反应4.5；Ba：外周血嗜碱性粒细胞比例；TKI：酪氨酸激酶抑制剂

3. TKI治疗结局及其影响因素分析：随访期内，324例（22.9％）发生治疗失败，其中107例（7.6％）疾病进展至加速期（52例，3.7％）或急变期（55例，3.9％），50例（3.6％）死亡，包括6例（0.4％）死于非CML原因、44例（3.2％）死于疾病进展。所有患者的7年FFS率为74.3％，PFS率为90.8％，OS率为95.2％，CML-OS率为95.7％。

为预测患者TKI治疗结局，运用ROC法对连续变量包括初诊时WBC、HGB、外周血嗜碱性粒细胞比例选取最大约登指数所对应值为截断值，取整后用于预测结局的截断值与预测治疗反应的截断值相同，即WBC 120.0×10^9^/L，HGB 115 g/L，外周血嗜碱性粒细胞比例10％。

为识别与结局相关的因素，单因素分析纳入因素同前文所述，多因素分析结果显示，受教育水平低、ELTS评分中/高危患者的治疗结局更差。另外，农村户籍、初诊时WBC≥120×10^9^/L、HGB<115 g/L是影响患者FFS的不利因素；初诊时HGB<115 g/L是影响患者PFS的不利因素；未婚及伴有合并症是影响患者OS及CML-OS的不利因素（表3）。

**表3:** 慢性髓性白血病（CML）慢性期患者结局的多因素分析结果

变量	FFS		PFS		OS		CML-OS	
	*HR*（95％*CI*）	*P*值	*HR*（95％*CI*）	*P*值	*HR*（95％*CI*）	*P*值	*HR*（95％*CI*）	*P*值
女性	0.8（0.6～1.0）	0.069						
婚姻状态						0.026		0.086
未婚（参考组）								
已婚					0.4（0.2～0.8）	0.008	0.4（0.2～0.9）	0.028
离异/丧偶					0.3（0.1～1.5）	0.144	0.4（0.1～2.0）	0.275
农村户籍	1.3（1.0～1.7）	0.034						
受教育水平		0.001		0.011		0.004		0.011
大学及以上（参考组）								
高中	1.3（1.0～1.8）	0.062	1.6（1.0～2.7）	0.053	1.7（0.8～3.9）	0.181	1.7（0.7～3.9）	0.235
初中及以下	1.7（1.3～2.2）	<0.001	1.9（1.2～3.0）	0.003	3.2（1.6～6.5）	0.001	3.0（1.5～6.3）	0.003
ELTS评分		<0.001		<0.001		<0.001		<0.001
低危（参考组）								
中危	1.8（1.3～2.3）	<0.001	2.1（1.3～3.4）	0.002	1.9（1.0～3.7）	0.069	1.6（0.8～3.4）	0.179
高危	2.9（2.0～4.0）	<0.001	4.9（2.9～8.2）	<0.001	5.4（2.7～10.8）	<0.001	5.1（2.5～10.6）	<0.001
WBC≥120×10^9^/L	1.3（1.0～1.8）	0.052						
HGB≥115 g/L	0.6（0.4～0.8）	<0.001	0.6（0.4～1.0）	0.034				
Ba≥10％								
有合并症					1.9（1.1～3.5）	0.031	1.8（1.0～2.5）	0.052
高危附加染色体异常								
服用仿制药								
一线二代TKI治疗								

注：FFS：无失败生存；PFS：无进展生存；OS：总生存；CML-OS：CML相关生存；Ba：外周血嗜碱性粒细胞比例；TKI：酪氨酸激酶抑制剂

4. 联合社会人口学因素与临床因素建立治疗反应预测模型：根据多因素分析识别出的治疗反应独立影响因素（性别、受教育水平、ELTS评分、初诊时WBC和HGB水平）及其*HR*权重对治疗反应获得的可能性赋值：女性、≥大学、ELTS评分低危、WBC<120×10^9^/L、HGB≥115 g/L各赋0分；男性、<大学、ELTS评分中危、WBC≥120×10^9^/L、HGB<115 g/L各赋1分；ELTS评分高危赋2分。根据上述赋分规则，将可评估细胞遗传学及分子学反应的1384例患者分为0分（90例，6.5％）、1分（321例，23.2％）、2分（328例，23.7％）、3分（268例，19.4％）、4分（226例，16.3％）、5分（108例，7.8％）和6分（43例，3.1％）组。由于0分组与1分组、3分组与4分组患者间治疗反应获得率差异无统计学意义，因此将其合并。最终，将患者分为5个亚组：极低危组（0～1分，411例，29.7％）、低危组（2分，328例，23.7％）、中危组（3～4分，494例，35.7％）、高危组（5分，108例，7.8％）、极高危组（6分，43例，3.1％），各组间7年累积CCyR（98.2％、95.4％、92.0％、85.1％、76.6％，*P*<0.001）、MMR（95.9％、87.8％、82.3％、74.5％、49.1％，*P*<0.001）、MR^4^（70.7％、63.3％、45.8％、25.5％、12.1％，*P*<0.001）和MR^4.5^（68.9％、57.6％、44.8％、22.7％、9.0％，*P*<0.001）获得率差异均有统计学意义（图1）。

**图1:**
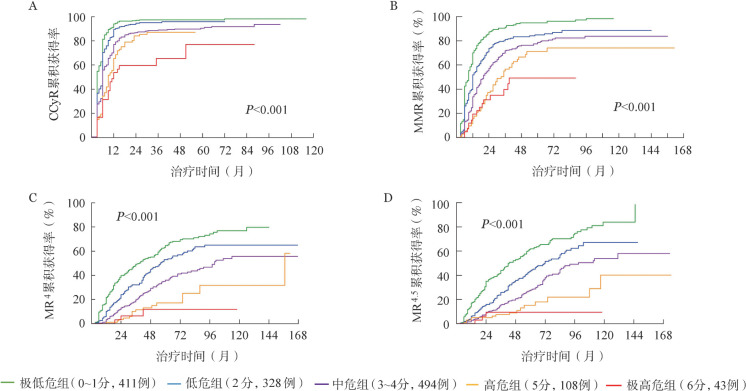
联合社会人口学因素及临床因素预测慢性髓性白血病慢性期患者酪氨酸激酶抑制剂治疗反应 A：预测完全细胞遗传学反应（CCyR）；B：预测主要分子学反应（MMR）；C预测分子学反应4（MR^4^）；D：预测分子学反应4.5（MR^4.5^）

5. 联合社会人口学因素与临床因素建立治疗结局预测模型：根据多因素分析识别出的治疗失败及疾病进展独立影响因素（受教育水平、ELTS评分和HGB水平）及其*HR*权重对治疗失败、疾病进展的可能性赋值：≥大学、ELTS评分低危、HGB≥115 g/L各赋0分；<大学、ELTS评分中危、HGB<115 g/L各赋1分；ELTS评分高危赋2分。最终，将所有1414例患者分为5个亚组：极低危组（0分，343例，24.3％）、低危组（1分，484例，34.2％）、中危组（2分，348例，24.6％）、高危组（3分，177例，12.5％）、极高危组（4分，62例，4.4％），各组间7年FFS率（92.0％、80.4％、67.5％、51.4％、30.2％，*P*<0.001）和PFS率（98.4％、93.5％、88.6％、78.4％、62.4％，*P*<0.001）差异均有统计学意义（图2）。

**图2:**
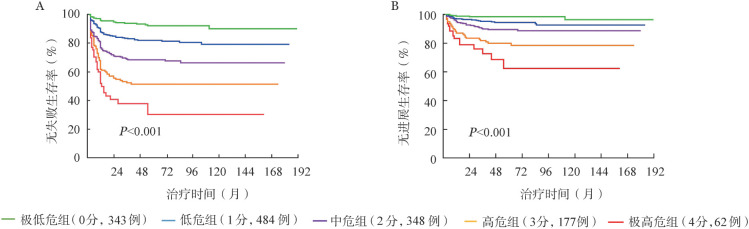
联合社会人口学因素及临床因素预测慢性髓性白血病慢性期患者无失败生存率（A）和无进展生存率（B）

根据多因素分析识别出的OS及CML-OS独立影响因素（受教育水平、婚姻状态、ELTS评分和合并症）及其*HR*权重对OS、CML-OS的可能性赋值：≥大学、已婚状态、ELTS评分低危、无合并症各赋0分；<大学、未婚或离异/丧偶、ELTS评分中危、有合并症各赋1分；ELTS评分高危赋2分。根据上述赋分规则，全部患者被分为0分（528例，37.3％）、1分（300例，21.2％）、2分（248例，17.5％）、3分（242例，17.1％）、4分（86例，6.1％）、5分（10例，0.8％）组。由于0分组与1分组、2分组与3分组、4分组与5分组患者间OS及CML-OS率差异无统计学意义，因此将其合并。最终，将总体患者分为3个亚组：低危组（0～1分，828例，58.6％）、中危组（2～3分，490例，34.6％）、高危组（4～5分，96例，6.8％），三组间7年OS率（97.8％、89.2％、70.6％，*P*<0.001）和CML-OS率（97.7％、90.4％、70.9％，*P*<0.001）差异均有统计学意义（图3）。

**图3:**
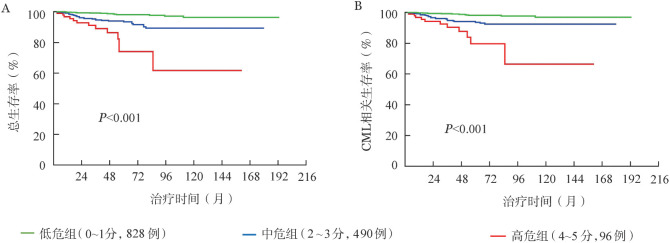
联合社会人口学因素及临床因素预测慢性髓性白血病（CML）慢性期患者总生存率（A）及CML相关生存率（B）

## 讨论

本研究回顾性分析1414例18～83岁初发CML-CP连续病例，探索影响患者TKI治疗反应及结局的社会人口学及临床因素，并联合社会人口学因素与临床因素预测患者TKI治疗反应及结局。本研究结果显示，男性、受教育水平低、初诊时WBC≥120×10^9^/L、HGB<115 g/L、ELTS评分中/高危和一线伊马替尼治疗与较低的细胞遗传学反应和分子学反应获得率均显著相关，农村户籍还与较低的CCyR获得率显著相关；受教育水平低、ELTS评分中/高危患者的治疗结局更差，农村户籍、初诊时WBC≥120×10^9^/L、HGB<115 g/L是影响FFS的不利因素；初诊时HGB<115 g/L是影响PFS的不利因素；未婚及伴有合并症是影响OS及CML-OS的不利因素。联合社会人口学因素及临床因素分别建立针对预测治疗反应、治疗失败及疾病进展、生存结局的三个新预测模型，均可成功预测各组间患者治疗反应及结局情况。

目前，绝大部分预测TKI治疗反应及结局的因素是基于患者临床特征，如Sokal、Hasford、EUTOS、ELTS评分、初诊时WBC、HGB、合并症、转录本类型、高危附加染色体异常等[28]–[35]。数项研究表明，除临床特征外，社会人口学因素也与预后显著相关[15]–[21]。一项来自印度的研究发现，低收入、自由职业及长期接触有毒物质如重金属、农药、辐射等环境因素的患者比高收入人群更容易被诊断出患有CML，且OS率往往较低[16]。一项来自美国的研究也发现，美国CML患者的OS率在低收入人群、男性及未婚患者显著降低[15]。一项英国的研究也得到了相似的结论：贫困地区CML患者的死亡风险是高收入地区患者的3倍以上[17]。但也有瑞典的研究显示，收入、受教育水平及居住环境等可能影响CML患者治疗选择，但并不影响患者OS结局[21]。近期，本中心报道中国CML患者受教育水平低、ELTS评分中/高危、初诊时WBC高、HGB低、一线伊马替尼治疗与其较低的治疗反应获得率相关；另外，受教育水平低、未婚状态、ELTS评分中/高危、初诊时HGB低与其较差的结局之间显著相关[22]。因此，除临床特征外，患者社会人口学特征也是不容忽视的影响TKI治疗反应及结局的重要因素。本次研究发现，患者初诊时伴有合并症与较差的生存结局显著相关，分析其可能的原因，初诊时伴有合并症可能影响患者药物选择及治疗依从性等，从而影响其治疗反应和结局。本研究还发现患者婚姻状况也显著影响其生存结局，可能的原因是单身或离婚/丧偶的患者缺乏家人的情感支持及治疗、监测依从性监督，已有相关文献报道独居患者依从性较差且监测频率较低，从而影响其治疗反应及结局[22],[36]–[38]。

患者受教育水平是社会人口学因素中对治疗反应及结局最为显著的共同影响因素。受教育水平较低的患者对于疾病的认知、自身健康状况以及对治疗、监测依从性的重视程度往往不够，患者在治疗过程中，自行减少TKI剂量、甚至停药，未定期复查、未及时监测并合理处理药物不良反应等, 导致治疗效果不佳甚至疾病进展, 从而影响治疗反应和结局[20]。对于高危与极高危患者来说，正确选择一线治疗药物的同时，还应加强对患者的疾病教育，进一步提升患者对疾病的认识水平、给患者提供更多的医疗保健信息与教育，以提高患者对治疗的依从性及对自身健康状况的重视程度，才能进一步改善患者整体结局。

本研究有以下局限性：①为回顾性研究；②纳入研究的部分患者采用一线二代TKI治疗；③本次研究中，尽管我们纳入了“户籍”这一社会人口学因素，但因属于单中心研究，纳入人群仅限于在我院就诊并接受治疗的患者，使分析结果可能产生偏倚；④某些社会人口学信息如受教育程度、婚姻状态等在疾病诊断后、随访过程中可能发生变化；⑤不能确认患者的服药依从性；⑥少数患者未进行规律监测。这些因素对研究结果可能产生一定影响。

总之，除临床特征外，社会人口学因素与CML患者治疗反应及结局密切相关。联合社会人口学因素与临床因素可更好地预测患者TKI治疗反应及结局。临床医师在管理CML患者时，应重视患者的社会人口学特征，识别容易被忽视的高危人群，予以更多的关注、教育和管理，帮助改善患者的整体治疗结局。
